# Treatment Patterns and Survival Outcomes of Non-Small Cell Lung Cancer Patients Initially Diagnosed With Brain Metastases in Real-World Clinical Practice

**DOI:** 10.3389/fonc.2020.581729

**Published:** 2020-10-09

**Authors:** Xin-Ru Chen, Xue Hou, Xiao-Xiao Dinglin, Yong-Dong Liu, Yin Li, Wei Zheng, De-Lan Li, Jing Chen, Xiao-Liang Wu, Kai-Cheng Wang, Shu-Xiang Ma, Yin-Duo Zeng, Li-Kun Chen

**Affiliations:** ^1^State Key Laboratory of Oncology in South, China Department of Medical Oncology, Sun Yat-Sen University Cancer Center, Collaborative Innovation Center for Cancer Medicine, Guangzhou, China; ^2^Guangdong Provincial Key Laboratory of Malignant Tumor Epigenetics and Gene Regulation, Breast Tumor Center, Sun Yat-Sen Memorial Hospital, Sun Yat-sen University, Guangzhou, China; ^3^Department of Pathology, The First Affiliated Hospital of Sun Yat-sen University, Guangzhou, China; ^4^Department of Endoscopy, Sun Yat-Sen University Cancer Center, State Key Laboratory of Oncology in South China, Collaborative Innovation Center for Cancer Medicine, Guangzhou, China; ^5^Department of Chemotherapy, Zhongshan City People’s Hospital, Zhongshan, China; ^6^Department of Oncology, Guizhou Provincial People’s Hospital, Guiyang, China; ^7^Department of Oncology, Affiliated Cancer Hospital of Zhengzhou University, Henan Cancer Hospital, Zhengzhou, China

**Keywords:** non-small-cell lung cancer, brain metastases, systemic medication, brain radiotherapy, survival outcomes

## Abstract

**Background:**

This study aimed to comprehensively analyze the characteristics, treatment patterns, and survival outcomes of non-small-cell lung cancer (NSCLC) patients initially diagnosed with brain metastases (BMs) in real-world practice.

**Methods:**

We enrolled NSCLC patients initially diagnosed with BMs between Jan 2004 and Jan 2018 in our institution. Patient demographics, treatment modalities, and survival outcomes were then analyzed. Brain localized treatment (BLT) included early brain radiotherapy (EBR), deferred brain radiotherapy (DBR), and surgery.

**Results:**

A total of 954 patients were identified. Concerning initial treatment, 525 patients (55.0%) received systemic medication (SM)+BLT, 400 patients (41.9%) received SM only, and 29 patients received BLT only (3.0%). SM+BLT cohort was associated with longer median overall survival (mOS) than the SM only and the BLT only cohorts both in epidermal growth factor receptor (EGFR)/anaplastic lymphoma kinase (ALK)-negative/unknown patients (15.3 months, 95% confidence interval [CI], 14.2–16.4; 11.1 months, 9.0–13.2; 7.0 months, 5.4–8.6; p<0.001) and in EGFR/ALK-positive patients (33.7 months, 28.5–38.9; 22.1 months, 17.8–26.4; 4.0 months, 3.6–4.4; p < 0.001). As for timing of radiotherapy, SM+EBR (14.1 months, 12.7–15.5) was associated with inferior mOS than SM+DBR (19.4 months, 14.2–24.6) in EGFR/ALK-negative/unknown patients. No significant difference was found in EGFR/ALK-positive patients (28.3 months, 19.1–37.5; 33.3 months, 28.1–38.5). Patients in the EGFR/ALK-negative/unknown cohort treated with first-line pemetrexed with platinum (PP) (15.8 months, 14.0–17.6, p<0.001) had longer mOS than those received non-PP regimens (13.1 months, 11.6–14.6). However, no difference was observed among EGFR/ALK-positive patients who were treated with tyrosine kinase inhibitors (TKIs) (29.5 months, 21.1–37.9; p = 0.140), PP (27.2 months, 21.6–32.8) and non-PP regimens (25.0 months, 16.0–34.0).

**Conclusions:**

Our study confirmed that the use of SM+BLT is associated with superior mOS than those treated with SM only and BLT only. SM+DBR might be a better radiotherapeutic strategy for this patient population. EGFR/ALK-negative/unknown patients showed a survival benefit with PP treatment.

## Introduction

Non-small-cell lung cancer (NSCLC) accounts for 80 to 85% of all lung cancers, and more than three-quarters of NSCLC patients present with advanced disease (1, 2). Brain is one of the most common sites of NSCLC metastases, and 30 to 50% of these patients may develop brain metastases (BMs) at some point in their lives (3, 4). Besides, 25–30% of patients will experience synchronous BMs (5). Though significant advances have been made in the management of NSCLC in recent years, the prognosis of patients with BMs is still poor.

The management of BMs usually requires a multidisciplinary team that integrates brain localized treatment (BLT), systemic medication (SM), and supportive care. The particular treatment option chosen for BMs mainly depends on the number of BMs, the presence of neurological symptoms, the presence of extracranial metastasis (ECM), the patient’s general health condition, and other factors (6). The current treatment options for managing BMs include surgical resection, whole-brain radiation therapy (WBRT), and stereotactic radiosurgery (SRS), either alone or combined with SM, such as chemotherapy or targeted therapy (7). Thus far, increasing numbers of studies have shown that SM has excellent efficacy for BMs, especially with the emergence of tyrosine kinase inhibitors (TKIs) that target epidermal growth factor receptor (EGFR) and anaplastic lymphoma kinase (ALK); patients with BMs who harbored these gene mutations thus have opportunities to forego local treatment to the brain in early days (8). Brain radiotherapy is a standard treatment for BMs, and various studies have explored the best sequence in which radiotherapy and SM should be administered in the context of newly diagnosed BMs (9–12). However, the results of these studies have been inconsistent.

Despite the high rate of BMs in NSCLC, patients initially diagnosed with BMs are commonly excluded in several prospective clinical trials, and thus, evidence-based data on the management of these patients are limited. Therefore, we performed a real-world study to comprehensively analyze the characteristics, treatment patterns, and survival outcomes in NSCLC patients with newly diagnosed BMs at a single institution in China.

## Materials and Methods

### Patients and Populations

This study was reviewed and approved by the Guangdong Association Study of Thoracic Oncology (No. A2017-002) and the institutional review board/ethics committee of the participating hospitals, and exception to the requirement of informed consent was approved. We retrospectively identified NSCLC patients who were initially diagnosed with BMs from Jan 2004 to Jan 2018. The inclusion criteria included the following: 1) histologically or cytologically confirmed NSCLC, 2) magnetic resonance imaging (MRI) scans of the brain identified BMs at the time of diagnosis, and 3) available clinical data and follow-up information at Yat-Sen University Cancer Center (SYSUCC). Patients who did not receive further treatment at our center after diagnosis and who did not have follow-up data were excluded. Patient follow-up concluded on October 10, 2019, thereby ensuring a minimum follow-up time of over 1 year for each patient.

The following characteristics were collected for analysis: age, gender, smoking history, Karnofsky Performance Status (KPS) at diagnosis, histology, EGFR and ALK rearrangement, number of BM, neurological symptoms, lungmol-GPA (Graded Prognostic Assessment) index, types of BLT, and name of SM. In our study, BLT included brain radiotherapy (WBRT, SRS, or a combination of the two) and brain surgery (BS). Deferred brain radiotherapy (DBR) was defined as patients who received initial brain radiotherapy after progression on first-line SM but otherwise was considered to be early brain radiotherapy (EBR).

### Statistical Analysis

Median overall survival (mOS) was calculated from the date of diagnosis of NSCLC with BMs to the date of death from any cause or the date of the last follow-up. OS was estimated using the Kaplan-Meier method and was compared using the log-rank test. Statistical differences in patient characteristics based on treatment patterns were analyzed using chi-square tests or Fisher’s exact test for categorical measures. To decrease potential treatment selection bias, propensity score matching (PSM) was conducted using a nearest-neighbor algorithm to adjust for demographical and clinical covariates. A two-sided p-value < 0.05 was considered statistically significant. All data were processed with SPSS version 22.0 (SPSS Inc., Chicago, IL, USA).

## Results

### Baseline Patient Characteristics

**Figure 1:**
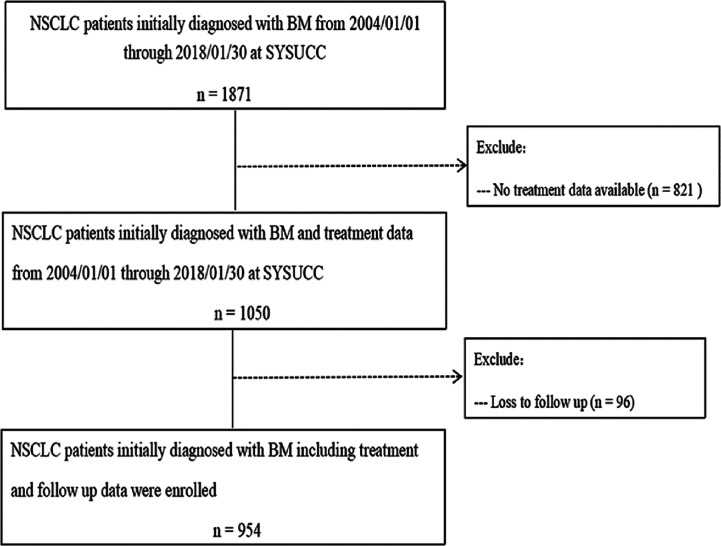
Patient flow chart illustrating selection of the study population. BMs, brain metastases; NSCLC, non-small cell lung cancer; SYSUCC, Sun Yat-Sen University Cancer Center.

In all, 954 patients were eligible for inclusion in our study (**Figure 1**). Regarding initial treatment approaches, 525 patients (55.0%) received SM+BLT, 400 patients (41.9%) received SM only, and 29 patients received BLT only (3.0%). As for BLT, 346 patients received 346 (36.3%) were treated with SM+EBR, 101 (10.6%) received SM+DBR, 78 (8.2%) received SM+BS. Patient characteristics are listed in **Table 1**. Due to the limited prevalence of testing technology, EGFR and ALK status was available in 619 (64.8%) patients from 2006 and 320 (33.5%) patients from 2012. Among them, 295 patients (41.8%) were EGFR mutant-positive and 27 patients (8.4%) were ALK rearrangement-positive.

**Table 1:** Baseline patient characteristics (n = 954).

Characteristic	No. (%)
Age, years Median (range) <65 ≥65	56 (17–89) 780 (81.8) 174 (18.2)
Gender Male Female	598 (62.7) 356 (37.3)
Smoking status Never Former/current	536 (56.2) 418 (43.8)
Pretreatment KPS ≤70 >70	81 (8.5) 873 (91.5)
Histology Adenocarcinoma Squamous carcinoma Others	839 (88.0) 70 (7.3) 45 (4.7)
No. of brain metastases 1 2–4 >4	319 (33.4) 203 (21.3) 432 (45.3)
Neurological symptoms Yes No	306 (32.1) 648 (67.9)
Extracranial metastases Yes No	538 (56.4) 416 (43.6)
Gene status EGFR mutation ALK rearrangement Wild type Unknown	295 (31.0) 27 (2.8) 297 (31.1) 335 (35.1)
Lung-molGPA* 0–1 1.5–2 2.5–3 3.5–4	92 (9.6) 346 (36.3) 406 (42.6) 110 (11.5)
Initial treatment SM only SM+EBR SM+DBR SM+BS BLT only	400 (41.9) 346 (36.3) 101 (10.6) 78 (8.2) 29 (3.0)

ALK, anaplastic lymphoma kinase; AP, pemetrexed with platinum; BS, brain surgery; DBR, deferred brain radiotherapy; DP, docetaxel with platinum; EBR, early brain radiotherapy; EGFR, epidermal growth factor receptor; GP, gemcitabine with platinum; GPA, graded prognostic assessment; KPS, Karnofsky performance status; SM, systemic medication; TKI, tyrosine kinase inhibitor; TP, paclitaxel with platinum.

*An update of the DS-GPA using molecular markers.

The baseline characteristics of 632 patients with negative/unknown EGFR/ALK status and 322 patients with positive EGFR/ALK status grouped according to five treatment patterns are listed in **Tables 2**, **3**. In the EGFR/ALK-negative/unknown cohort, SM only was more likely to be administered to patients with no neurological symptoms (83.6%), those with extracranial metastases (67.2%), and those with a Lung-molGPA of 0–2 (72.7%). SM+EBR was more likely to be administered to patients with >4 brain metastases (51.1%), those presenting with neurological symptoms (64.8%), and those with a lung-molGPA of 0–2 (60.9%). SM+DBR was more likely to be administered to patients without neurological symptoms (77.8%) and to those with a lung-molGPA of 0–2 (66.7%). SM+BS was more likely to be administered to patients with one brain lesion (78.4%), those with symptomatic BMs (84.3%), those without extracranial metastases (86.3%), and those with a favorable prognosis with a lung-molGPA of 2.5–3 (68.6%). Finally, BLT only was more likely to be administered to patients with >4 brain metastases (75.0%), those presenting with neurological symptoms (75.0%), and those with a lung-molGPA of 0–2 (75.0%). In patients with positive EGFR/ALK status, the characteristics of patients who received SM+BS or BLT only were comparable to those with negative/unknown EGFR/ALK status but were somewhat different from the other three patterns. SM only was more likely to be administered to patients with a lung-molGPA of 2.5–4 (92.0%), while SM+EBR was more likely to be administered to patients without neurological symptoms (55.6%) and those with a lung-molGPA of 2.5–4 (87.8%). SM+DBR was more likely to be administered to patients with a lung-molGPA of 2.5–4 (86.8%). Consistently, patients who received EBR were more likely to have >4 BMs compared with patients who received DBR either in the negative/unknown cohort (51.1 *vs.* 34.9%) or in the positive cohort (62.2 *vs.* 44.8%).

**Table 2:** Characteristics of the 632 EGFR/ALK-negative/unknown patients.

Characteristic	SM only (n = 238)	SM+EBR (n = 256)	SM+DBR (n = 63)	SM+BS (n = 51)	BLT only (n = 24)	p-value
	No. (%)	No. (%)	No. (%)	No. (%)	No. (%)	
Age, years Median (range) <65 ≥65	57 (24–81) 186 (78.2) 52 (21.8)	56 (24–80) 209 (81.6) 47 (19.4)	55 (24–77) 52 (82.5) 11 (17.5)	57 (27–79) 39 (76.5) 12 (23.5)	57 (30–74) 15 (62.5) 9 (37.5)	0.213
Gender Male Female	161 (67.6) 77 (32.4)	186 (78.2) 70 (11.8)	42 (66.7) 20 (33.3)	41 (80.4) 10 (19.6)	20 (83.3) 4 (16.7)	0.216
Smoking status Never Former/current	115 (48.3) 123 (51.7)	113 (47.5) 143 (52.5)	35 (55.6) 28 (44.4)	26 (51.0) 25 (49.0)	11 (45.8) 13 (54.2)	0.537
Pretreatment KPS >70 ≤70	211 (88.7) 27 (11.3)	232 (90.6) 24 (9.4)	56 (88.9) 7 (11.1)	45 (88.2) 6 (11.8)	18 (75.0) 6 (25.0)	0.235
Histology Adenocarcinoma Non-adenocarcinoma	202 (84.9) 36 (15.1)	213 (83.2) 43 (16.8)	57 (90.5) 6 (9.5)	38 (74.5) 13 (25.5)	22 (91.7) 2 (8.3)	0.178
No. of brain metastases 1 2–4 >4	92 (38.7) 49 (20.6) 97 (40.7)	69 (27.0) 56 (21.9) 131 (51.1)	26 (41.3) 15 (23.8) 22 (34.9)	40 (78.4) 3 (5.9) 8 (15.7)	4 (16.7) 2 (8.3) 18 (75.0)	<0.001
Neurological symptoms Yes No	39 (16.4) 199 (83.6)	166 (64.8) 90 (35.2)	14 (22.2) 49 (77.8)	43 (84.3) 8 (15.7)	18 (75.0) 6 (25.0)	<0.001
Extracranial metastases Yes No	160 (67.2) 78 (32.8)	129 (50.4) 127 (49.6)	37 (58.7) 26 (41.3)	7 (13.7) 44 (86.3)	10 (41.7) 14 (58.3)	<0.001
Lung-molGPA* 0–2 2.5–3	173 (72.7) 65 (27.3)	156 (60.9) 100 (39.1)	42 (66.7) 21 (33.3)	16 (31.4) 35 (68.6)	18 (75.0) 6 (25.0)	<0.001
First-line chemotherapy PP TP DP GP Others	115 (48.3) 81 (34.0) 19 (8.0) 13 (5.5) 10 (4.2)	92 (35.9) 101 (39.5) 24 (9.4) 23 (9.0) 16 (6.2)	26 (41.3) 20 (31.7) 3 (4.8) 8 (12.7) 6(9.5)	31 (60.8) 13 (25.5) 1 (2.0) 1 (2.0) 5 (9.7)	/ / / / /	
Brain localized therapy WBRT SRS WBRT+SRS Surgery	/ / / /	183 (71.5) 50 (19.5) 23 (9.0) /	52 (82.5) 9 (14.3) 2 (3.2) /	/ / / 51 (100.0)	9 (37.5) 3 (12.5) 1 (4.2) 11 (45.8)	

ALK, anaplastic lymphoma kinase; BLT, brain localized treatment; BS, brain surgery; DBR, deferred brain radiotherapy; DP, docetaxel with platinum; EBR, early brain radiotherapy; EGFR, epidermal growth factor receptor; GP, gemcitabine with platinum; GPA, graded prognostic assessment; KPS, Karnofsky performance status; PP, pemetrexed with platinum; SRS, stereotactic radiosurgery; SM, systemic medication; TP, paclitaxel with platinum; WBRT, whole-brain radiotherapy.

*An update of the DS-GPA using molecular markers.

**Table 3:** Characteristics of the 322 EGFR/ALK-positive patients.

Characteristic	SM only (n = 162)	SM+EBR (n = 90)	SM+DBR (n = 38)	SM+BS (n = 27)	BLT only (n = 5)	p-value
	No. (%)	No. (%)	No. (%)	No. (%)	No. (%)	
Age, years Median (range) <65 ≥65	53 (26–89) 138 (85.2) 24 (14.8)	54 (17–78) 79 (87.8) 11 (12.2)	53 (33–67) 37 (97.4) 1 (2.6)	61 (29–74) 21 (77.8) 6 (22.2)	50 (49–70) 4 (80.0) 1 (20.0)	0.106
Gender Male Female	70 (43.2) 92 (56.8)	45 (50.0) 45 (50.0)	21 (55.3) 17 (44.7)	10 (37.0) 17 (63.0)	1 (20.0) 4 (80.0)	0.345
Smoking status Never Former/current	125 (77.2) 37 (22.8)	57 (63.3) 33 (36.7)	25 (65.8) 13 (34.2)	24 (88.9) 3 (11.1)	5 (100.0) 0 (0)	0.019
Pretreatment KPS >70 ≤70	155 (95.7) 7 (4.3)	88 (97.8) 2 (2.2)	38 (100.0) 0 (0)	26 (96.3) 1 (3.7)	4 (80.0) 1 (20.0)	0.212
Histology Adenocarcinoma Non-adenocarcinoma	156 (96.3) 6 (3.7)	86 (95.6) 4 (4.4)	37 (97.4) 1 (2.6)	24 (88.9) 3 (11.1)	4 (80.0) 1 (20.0)	0.166
No. of brain metastases 1 2–4 >4	48 (29.6) 41 (25.3) 73 (45.1)	18 (20.0) 16 (17.8) 56 (62.2)	7 (18.4) 14 (36.8) 17 (44.8)	14 (51.9) 7 (25.0) 6 (22.1)	1 (20.0) 0 (0) 4 (80.0)	0.003
Neurological symptoms No Yes	25 (15.4) 137 (84.6)	40 (44.4) 50 (55.6)	9 (23.7) 29 (76.3)	24 (88.9) 3 (11.1)	4 (80.0) 1 (20.0)	<0.001
Extracranial metastases Yes No	106 (65.4) 56 (34.6)	52 (57.8) 38 (42.2)	28 (73.7) 10 (26.3)	20 (74.1) 7 (25.9)	2 (40.0) 3 (60.0)	0.225
Gene status EGFR mutation ALK rearrangement	154 (95.1) 8 (4.9)	82 (91.1) 8 (8.9)	34 (89.5) 4 (10.5)	22 (81.5) 5 (18.5)	3 (60.0) 2 (40.0)	0.015
Lung-molGPA* 0–2 2.5–4	13 (8.0) 149 (92.0)	11 (12.2) 79 (87.8)	5 (13.2) 33 (86.8)	2 (8.0) 25 (92.0)	2 (40.0) 3 (60.0)	0.165
First-line chemotherapy EGFR TKIs ALK TKIs PP TP Others	79 (48.8) 2 (1.2) 72 (44.4) 8 (4.0) 1 (0.6)	43 (47.8) 2 (2.2) 27 (30.0) 10 (11.1) 8 (4.9)	21 (55.3) 2 (5.3) 14 (36.8) 1 (2.6) 0 (0)	15 (55.6) 1 (3.7) 10 (37.0) 0 (0) 1 (3.7)	/ / / / /	
Brain localized therapy WBRT SRS WBRT+SRS Surgery	/ / / /	64 (71.1) 21 (23.3) 5 (5.6) /	34 (89.4) 2 (5.3) 2 (5.3) /	/ / / 27 (100.0)	3 (60.0) 0 (0) 0 (0) 2 (40.0)	

ALK, anaplastic lymphoma kinase; BLT, brain localized treatment; BS, brain surgery; DBR, deferred brain radiotherapy; EBR, early brain radiotherapy; EGFR, epidermal growth factor receptor; GPA, graded prognostic assessment; KPS, Karnofsky performance status; PP, pemetrexed with platinum; SRS, stereotactic radiosurgery; SM, systemic medication; TKI, tyrosine kinase inhibitor; TP, paclitaxel with platinum; WBRT, whole-brain radiotherapy.

*An update of the DS-GPA using molecular markers.

### Treatment Patterns and Survival Outcomes

In the entire population, the median follow-up time was 54.8 months (interquartile range, 9.0–33.0). The mOS was 16.5 months (95% confidence intervals [CI], 15.4–17.6). The OS at 1, 3, and 5 years was 64.4, 22.0, and 9.2%, respectively.

**Figure 2:**
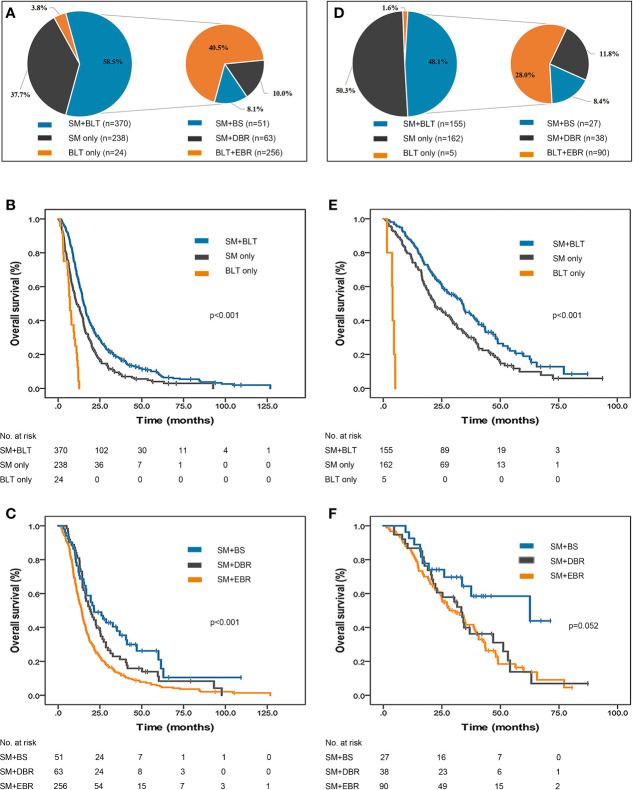
Treatment patterns and survival outcomes of NSCLC patients initially diagnosed with BMs. **(A)** Distribution of treatment patterns in 632 EGFR/ALK-negative/unknown patients. The mOS of EGFR/ALK-negative/unknown patients treated with SM only, **(B)** SM+BLT and BLT only. **(C)** The mOS of EGFR/ALK-negative/unknown patients treated with SM only, SM+EBR, SM+DBR, SM+BS, and BLT only. **(D)** Distribution of treatment patterns in 322 EGFR/ALK-positive patients. **(E)** The mOS of EGFR/ALK-positive patients treated with SM only, SM+BLT and BLT only. **(F)** The mOS of EGFR/ALK-positive patients treated with SM only, SM+EBR, SM+DBR, SM+BS, and BLT only. ALK, anaplastic lymphoma kinase; BLT, brain localized treatment; BMs, brain metastases; BS, brain surgery; DBR, deferred brain radiotherapy; EBR, early brain radiotherapy; EGFR, epidermal growth factor receptor; NSCLC, non-small cell lung cancer; mOS, median overall survival; SM, systemic medication.

The distribution of treatment patterns in 632 EGFR/ALK-negative/unknown patients is shown in **Figure 2**. More than half the patients received SM+BLT (370, 58.5%) followed by SM (238, 37.7%). Among those treated with SM+BLT, SM+EBR (256/370, 69.2%) was the most common treatment strategy. The mOS of the SM+BLT cohort was significantly longer (15.3 months, 95% CI, 14.2–16.4, p < 0.001, **Figure 2**) than SM only (11.1 months, 95% CI, 9.0–13.2) and BLT only cohorts (7.0 months, 95% CI, 5.4–8.6). Moreover, patients who received SM+EBR (14.1 months, 95% CI, 12.7–15.5, p < 0.001, **Figure 2**) had a poor mOS compared with patients who received SM+BS (21.5 months, 95% CI, 11.5–31.5) and SM+DBR (19.4 months, 95% CI, 14.2–24.6).

The distribution of treatment patterns in 322 EGFR/ALK-positive patients is presented in **Figure 2**. The percentage of the SM only (162, 50.3%) cohort was slightly higher than that of the SM+BLT (155, 48.1%) cohort. Besides, SM+EBR (90/155, 58.1%) remained the most common combined therapy strategy. SM+BLT (33.7 months, 95% CI, 28.5–38.9, p < 0.001, **Figure 2**) was associated with an improved mOS compared with SM only (22.1 months, 95% CI, 17.8–26.4) and BLT only (4.0 months, 95% CI, 3.6–4.4). Furthermore, the mOS was better in the SM+BS group (62.6 months, 95% CI, 12.4–112.7, p = 0.052, **Figure 2**) than in the SM+EBR (28.3 months, 95% CI, 19.1–37.5) and SM+DBR (33.3 months, 95% CI, 28.1–38.5) groups, but the difference was not statistically significant.

To clarify the population that would benefit from different combined treatments, we subdivided patients according to the lung-molGPA index. Patients with a more favorable prognosis (lung-molGPA 2.5–4) who received SM+BS had a longer mOS (40.6 months, 95% CI, 28.6–52.6, p < 0.001, **Supplementary Figure S1A**) than those who were treated with SM+EBR (22.0 months, 95% CI, 18.3–25.7) and those who received SM+DBR (30.3 months, 95% CI, 18.4–42.2); while those with a less favorable prognosis (lung-molGPA 0–2) did not demonstrate improved mOS when treated with SM+BS (15.2 months, 95% CI, 8.1–22.3, **Supplementary Figure S1B**) compared with those who received SM+EBR (12.7 months, 95% CI, 11.2–14.2) and SM+DBR (19.4 months, 95% CI, 13.4–25.4).

### Early Brain Radiotherapy and Deferred Brain Radiotherapy After Propensity Score Matching

To further investigate the optimal timing of radiotherapy for NSCLC patients initially diagnosed with BMs, we performed PSM to balance the clinical factors between the EBR group and the DBR group. After balancing the covariates, 63 matched patients from the SM+DBR group and 189 matched patients from the SM+EBR group out of the EGFR/ALK-negative/unknown patients were compared and analyzed (1:3 match). The results showed that they were well balanced (**Supplementary Table S1**). Consistent with the result without PSM, the mOS was also significantly improved in patients who received SM+DBR (19.4 months, 95% CI: 14.2–24.6, p = 0.015, **Supplementary Figure S2A**) compared with those who received SM+EBR (14.3 months, 95% CI: 12.8–15.8).

Besides, 38 matched EGFR/ALK-positive patients from the SM+DBR and the SM+EBR groups were compared and analyzed (1:1 match) (**Supplementary Table S2**). No significant difference in survival was observed between patients who received SM+DBR (30.8 months, 95% CI: 20.0–41.6, p = 0.846, **Supplementary Figure S2B**) and those treated with SM+EBR (33.3 months, 95% CI: 28.1–38.5).

### First-Line Systemic Medication and Survival Outcomes

**Figure 3:**
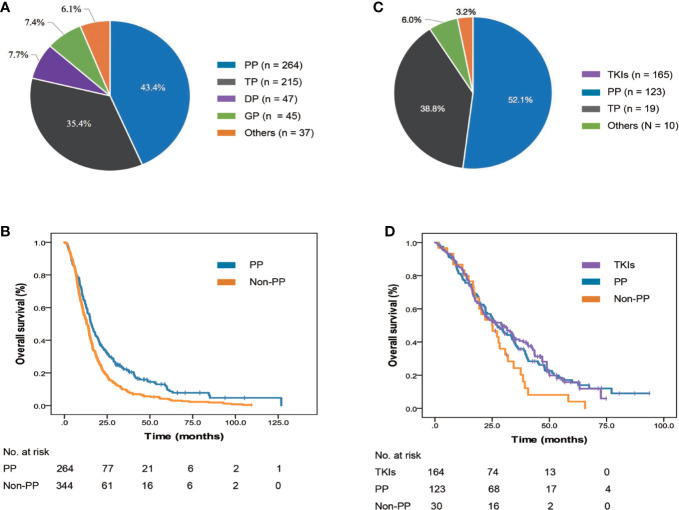
Frequency and survival outcomes of different systemic medications used as first-line regimens. **(A)** Frequency of treatment regimens in 632 EGFR/ALK-negative/unknown patients. **(B)** The mOS of EGFR/ALK-negative/unknown patients treated with AP and non-AP regimens. **(C)** Frequency of treatment regimens in 322 EGFR/ALK-positive patients. **(D)** The mOS of EGFR/ALK-positive patients treated with TKIs, AP, and non-AP regimens. ALK, anaplastic lymphoma kinase; DP, docetaxel with platinum; EGFR, epidermal growth factor receptor; GP, gemcitabine with platinum; mOS, median overall survival; PP, pemetrexed with platinum; TKI, tyrosine kinase inhibitor; TP, paclitaxel with platinum.

First-line systemic therapies were administered to 925 (97.0%) patients. For 608 patients with negative/unknown EGFR/ALK status, the most frequently used chemotherapy regimen was pemetrexed with platinum (PP; n = 264 patients, 43.4%, **Figure 3**). Patients who received PP (15.8 months, 95% Cl: 14.0–17.6, p < 0.001, **Figure 3**) were observed to have a longer mOS than those treated with non-PP regimens (13.1 months, 95% Cl: 11.6–14.6).

In the 317 patients with positive EGFR/ALK status, the most frequently used regimens in the first-line setting was EGFR/ALK TKIs (n = 164 patients, 51.7%, **Figure 3**). Among 157 patients received EGFR TKIs, all patients were treated with first-generation EGFR TKI, such as gefitinib, erlotinib, and icotinib, and no patients received osimertinib, the third generation EGFR TKI, which was granted accelerated approval by the China Food and Drug Administration (CFDA) in March 2017 to treat T790M-positive NSCLC patients as second-line therapy. For seven patients who received ALK TKIs, all were treated with crizotinib as first-line regimen. No significant difference was observed among patients who received EGFR/ALK TKIs (29.5 months, 95% Cl: 21.1–37.9, p = 0.140, **Figure 3**), PP (27.2 months, 95% Cl: 21.6–32.8) and non-PP regimens (25.0 months, 95% Cl: 16.0–34.0).

Besides, our study consisted of 88% of adenocarcinoma and 12% of non-adenocarcinoma, the detailed first-line treatment regimens subdivided by histology were shown in **Supplementary Table S3**.

## Discussion

The results of this study provide real-world experience in advanced NSCLC patients initially diagnosed with BMs, including treatment choices and survival outcomes, and help to provide an understanding of how the different treatment strategies that are currently available should be sequenced during the course of therapy. To our knowledge, this is the largest retrospective analysis of the literature on NSCLC patients initially diagnosed with BMs in China.

Our study indicated that SM combined with BLT was a better treatment strategy than SM or BLT alone regardless of EGFR and ALK status. Previous studies have described the poor ability of drugs to penetrate the blood-brain barrier (BBB), and considerable efforts have been made in developing novel agents that have better BBB penetration than earlier drugs (13, 14). Although newly developed drugs such as pemetrexed and EGFR/ALK TKIs have shown certain intracranial activity, many patients will experience progression of intracranial disease due to a lack of more effective drugs (15–18). Brain radiotherapy remains an important treatment option due to its simplicity of delivery and its ability to treat intracranial disease.

Though the QUARTZ study reported that compared with optimal supportive care, WBRT provided little additional clinical benefit for patients with BMs, approximately 40% of patients in this cohort had a KPS less than 70, and more than 50% of patients had an uncontrolled primary lung tumor (19). Additionally, enrolled patients were not eligible for SRS and surgery, which essentially caused selection bias in that patients with the poorest prognosis received WBRT. Comparatively, in our study, enrolled patients were newly diagnosed with BMs with good performance status, and several patients were also treated with SRS as well as with surgery. Therefore, brain localized therapies should not be omitted, as they might improve the survival and quality of life of some selected patients.

In our study, the impact of SM+BS on patient survival was most pronounced in patients with a more favorable prognosis (lung-molGPA 2.5–4), which can be explained by the nature of oligometastatic disease. More patients in this subgroup have neurological symptoms, 1–4 BMs and do not have extracranial metastasis (ECM). In addition, a subset of these patients also received aggressive treatment for primary lung lesions. Mounting evidence has suggested that aggressive treatment for oligometastatic NSCLC is needed, especially since the appropriate use of multidisciplinary methods may lead to a curable disease state and increased long-term survival (20). Therefore, for patients with oligometastatic BMs, BS could be used as an aggressive treatment option, while SM controls ECM and potentially controls intracranial micrometastatic disease.

Brain radiotherapy (WBRT and SRS) is the most widely used therapeutic approach in patients with BMs. In clinical practice, many clinicians may choose brain radiotherapy to alleviate neurological symptoms or as salvage radiotherapy after failure of standard treatment. However, the optimal timing of brain radiotherapy for NSCLC patients with BM remains controversial. Moreover, treatment strategies for BMs and extracranial lesions in NSCLC patients with wild-type genes or unknown genetics and those with oncogenic-driven NSCLC vary widely. In the current study, for patients with negative/unknown EGFR/ALK status, the proportion of patients who received SM+EBR was similar to those received SM but four times as high as those who received SM+DBR; it was also found that more patients in the SM+EBR group had neurological symptoms. This suggests that initial treatment with EBR was preferred by many practitioners for managing symptomatic BMs. However, patients who received DBR were more likely to have improved mOS relative to those who received EBR, and this result was unchanged after PSM. Consistent with a randomized phase III study conducted by Robinet, the results showed that the timing (early or delayed) of WBRT did not influence the survival of NSCLC patients with BMs who were treated with concurrent chemotherapy (21). Other prospective studies have also reported similar results and indicated that brain radiotherapy, especially early WBRT, may impair patients’ neurocognitive function (16, 22, 23).

For patients with positive EGFR/ALK status, the percentage of patients who received SM+EBR was also higher than the percentage of patients who received SM+DBR. With a sharp decline in the proportion of patients in the SM+EBR group compared with those in the EGFR/ALK-negative/unknown group, more clinicians chose SM alone as an initial treatment modality, which might be related to the higher efficacy of EGFR or ALK TKIs compared with traditionally cytotoxic drugs. Growing evidence suggests that EGFR or ALK TKIs, especially next-generation TKIs, might allow patients with BMs to forego local treatment to the brain, as they have already shown important activity for intracranial disease (24, 25). Until now, randomized studies on the optimal timing of brain radiotherapy in cases of oncogenic-driven NSCLC with BMs are scarce, and most studies were retrospective in nature. Results from three meta-analyses reported a similar survival after front-line EGFR TKI with brain RT (generally WBRT) and EGFR TKI alone (26–28). However, findings from another retrospective study conducted by Magnuson et al. showed a significantly worse survival in EGFR-mutated patients who received delayed brain radiotherapy, regardless of whether they underwent SRS or WBRT (29). Our study showed that patients who received SM+EBR failed to exhibit improved survival compared with those treated with SM+DBR, and the result was identical after PSM. But we should notice that most patients in these two groups were initially treated with WBRT, the sample size of patients who received SRS was too small. Therefore, whether the optimal timing of brain radiotherapy is related to different radiotherapy technologies remains needed to investigate and more randomized clinical trials are warranted. To our knowledge, several clinical trials (NCT02714010 and NCT02338011) are in progress and will compare the efficacy of upfront EGFR TKI *vs.* WBRT in EGFR-mutated NSCLC patients with newly diagnosed BMs.

Among patients with EGFR/ALK-negative/unknown status, those who received first-line PP exhibited a longer mOS than those who were treated with other platinum-based doublet agents. Notably, approximately 94.7% of patients who received PP regimen were adenocarcinoma. In a retrospective study, Yu et al. reported that BMs patients with EGFR wild-type or unknown status who received a combination of pemetrexed and platinum tended to have prolonged survival, with a mOS of 21 months (30). Consistent with the data reported by Sibilot M, the mOS of those treated with PP was as high as 9.3 months, which was longer than after other chemotherapy regimens (31). However, for patients in the EGFR/ALK-positive cohort, no survival benefit was identified among first-line TKIs, PP, and non-PP regimens. The main reason for this might be that a large proportion of patients who received first-line chemotherapy were administered TKIs as a second-line and above treatment.

Our study had some limitations. Firstly, this retrospective study had inherent biases. We excluded patients with missing medical records and those who were lost to follow-up, both of which could lead to selection bias. Secondly, due to the small sample size in some radiotherapy subgroups, we were unable to further compare the efficacies of different radiotherapy methods. Thirdly, this study was based on data from a top cancer center in China, which may not represent the real-world experiences of hospitals at other levels.

The current study provides a unique set of real-world data that adds to the current understanding of treatment decisions and survival outcomes in NSCLC patients initially diagnosed with BMs. Our study confirmed that patients who initially received SM+BLT are associated with superior mOS than those who were treated with SM only and BLT only. Besides, DBR might be a better timing of radiotherapy for this group of patients. EGFR/ALK-negative/unknown patients, especially for those with adenocarcinoma, can significantly benefit from chemotherapy consisting of pemetrexed plus platinum. In the future, prospective clinical trials and multidisciplinary participation will be required to clarify the optimal treatment pattern for NSCLC patients initially diagnosed with BMs.

## Data Availability Statement

All datasets presented in this study are included in the article/**Supplementary Material**.

## Ethics Statement

The studies involving human participants were reviewed and approved by Guangdong Association Study of Thoracic Oncology (No. A2017-002) and the institutional review board/ethics committee of the participating hospitals. Written informed consent for participation was not required for this study in accordance with the national legislation and the institutional requirements.

## Author Contributions

X-RC, XH, X-XD, and Y-DL had full access to all of the data in the study and takes responsibility for the integrity of the data and the accuracy of the data analysis. X-RC, XH, YL, WZ, D-LL, and JC contributed to the study design. X-LW, K-CW, S-XM, and Y-DZ contributed to data acquisition and critical history review. All authors contributed to the article and approved the submitted version.

## Funding

This work was supported by the National Natural Science Foundation of China (Grant No. 81572270), Sun Yat-Sen University Young Teacher Plan (Grant No. 19ykpy179), and Guangzhou Science and Technology Program (Grant No. 202002020074).

## Conflict of Interest

The authors declare that the research was conducted in the absence of any commercial or financial relationships that could be construed as a potential conflict of interest.

## Abbreviations

ALK, Anaplastic lymphoma kinase; BBB, Blood-brain barrier; BLT, Brain localized treatment; BMs, Brain metastases; BS, Brain surgery; CFDA, China Food and Drug Administration; CI, Confidence intervals; DBR, Deferred brain radiotherapy; DP, Docetaxel with platinum; EBR, Early brain radiotherapy; ECM, Extracranial metastasis; EGFR, Epidermal growth factor receptor; GP, Gemcitabine with platinum; GPA, Graded Prognostic Assessment; KPS, Karnofsky Performance Status; mOS, Median overall survival; MRI, Magnetic resonance imaging; NSCLC, Non-small-cell lung cancer; PP, Pemetrexed with platinum; PSM, Propensity score matching; SM, Systemic medication; SRS, Stereotactic radiosurgery; SYSUCC, Sun Yat-Sen University Cancer Center; TKIs, Tyrosine kinase inhibitors; TP, Paclitaxel with platinum; WBRT, Whole-brain radiation therapy.
