# Supplementary material for: Treatment Patterns and Survival Outcomes of Non-Small Cell Lung Cancer Patients Initially Diagnosed With Brain Metastases in Real-World Clinical Practice
Source: Front Oncol. 2020 Oct 9;10:581729. doi: 10.3389/fonc.2020.581729 (PMC7581726; doi:10.3389/fonc.2020.581729)
Supplement: Supplementary file 1 [file DataSheet_1.docx]

**Supplemental Data: Supplemental Table and** **Figure**

**Supplementary Online Content**

[Table S1 Characteristics of EBR and DBR groups in patients with negative/unknown EGFR/ALK status after propensity score matching (1:3) 2](#_Toc7851)

[Table S2 Characteristics of EBR and DBR groups in patients with positive EGFR/ALK status after propensity score matching (1:1) 3](#_Toc11558)

[Table S3 Table S3 Frequency of different systemic medications used as first-line regimens subdivide by histology.](#_Toc11558)......................................................................................................................4

[Figure S1. The OS of SM+BLT group stratified by Lung-molGPA. (A) Lung-molGPA 0-2; (B) Lung-molGPA 2.5-4. 5](#_Toc17764)

[Figure S2. Kaplan-Meier analysis comparing OS in patients treated with SM+EBR and SM+DBR after PSM. (A) In EGFR/ALK-negative/unknown cohort (1: 3 match). (B) In EGFR/ALK-positive cohort (1:1 match) 6](#_Toc30411)

**Table S1 Characteristics of EBR and DBR groups in patients with negative/unknownEGFR/ALK status after propensity score matching (1:3)**

| Characteristic | EBR (n=189) | DBR (n=63) | p-value |
| --- | --- | --- | --- |
|  | No. (%) | No. (%) |  |
| Age, years  <65  ≥65 | 159 (84.1)  30 (15.9) | 52 (82.5)  11 (17.5) | 0.844 |
| Gender  Male  Female | 134 (70.9)  55 (29.1) | 43 (68.3)  0 (31.7) | 0.751 |
| Smoking status  Never  Former/current | 92 (48.7)  97 (51.3) | 35 (55.6)  28 (44.4) | 0.384 |
| Pretreatment KPS  >70  ≤70 | 169 (89.4)  20 (10.6) | 56 (88.9)  7 (11.1) | 1.000 |
| Histology  Adenocarcinoma  Non-adenocarcinoma | 167 (88.4)  22 (11.6) | 57 (90.5)  6 (9.5) | 0.818 |
| No. of brain metastases  1-4  >4 | 112 (59.3)  77 (40.7) | 41 (65.1)  22 (34.9) | 0.458 |
| Neurological symptoms  Yes  No | 48 (25.4)  141 (74.6) | 14 (22.2)  49 (77.8) | 0.736 |
| Extracranial metastases  Yes  No | 114 (60.3)  75 (39.7) | 37 (58.7)  26 (41.3) | 0.882 |
| Brain radiotherapy  WBRT  SRS  WBRT+SRS | 148 (78.3)  34 (18.0)  7 (3.7) | 52 (82.5)  9 (14.3)  2 (3.2) | 0.868 |
| Abbreviations: ALK, anaplastic lymphoma kinase; DBR, deferred brain radiotherapy; EBR, early brain radiotherapy; EGFR, epidermal growth factor receptor; KPS, karnofsky performance status; SRS, stereotactic radiosurgery; WBRT, whole brain radiotherapy. | | | |

| Characteristic | EBR (n=38) | DBR (n=38) | p -value |
| --- | --- | --- | --- |
|  | No. (%) | No. (%) |  |
| Age, years  <65  ≥65 | 37 (97.4)  1 (2.6) | 37 (97.4)  1 (2.6) | 1.000 |
| Gender  Male  Female | 21 (55.3)  17 (44.7) | 22 (57.9)  16 (42.1) | 1.000 |
| Smoking status  Never  Former/current | 25 (65.8)  13 (34.2) | 25 (65.8)  13 (34.2) | 1.000 |
| Pretreatment KPS  >70  ≤70 | 38 (100.0)  0 (0) | 38 (100.0)  0 (0) | 1.000 |
| Histology  Adenocarcinoma  Non-adenocarcinoma | 37 (97.4)  1 (2.6) | 37 (97.4)  1 (2.6) | 1.000 |
| No. of brain metastases  1-4  >4 | 21 (55.3)  17 (44.7) | 15 (39.5)  23 (60.5) | 0.251 |
| Neurological symptoms  Yes  No | 29 (76.3)  9 (23.7) | 28 (73.7)  10 (26.3) | 1.000 |
| Extracranial metastases  Yes  No | 28 (73.7)  10 (26.3) | 32 (84.2)  6 (15.8) | 0.399 |
| Brain radiotherapy  WBRT  SRS  WBRT+SRS | 34 (89.5)  2 (5.3)  2 (5.3) | 32 (84.2)  4 (10.5)  2 (5.3) | 0.877 |
| Abbreviations: ALK, anaplastic lymphoma kinase; DBR, deferred brain radiotherapy; EBR, early brain radiotherapy; EGFR, epidermal growth factor receptor; KPS, karnofsky performance status; SRS, stereotactic radiosurgery; WBRT, whole brain radiotherapy. | | | |

**Table S2 Characteristics of EBR and DBR groups in patients with positive EGFR/ALK status after propensity score matching (1:1)**

**Table S3 Frequency of different systemic medications used as first-line regimens subdivide by histology.**

| EGFR/ALK-negative/unknown | Adenocarcinoma  (n = 510) | Squamous cell carcinoma  (n = 63) | Others  (n = 35) |
| --- | --- | --- | --- |
| PP | 250 (49.0) | 2 (3.2) | 12 (34.3) |
| TP | 154 (30.2) | 44 (69.8) | 17 (48.6) |
| DP | 37 (7.3) | 6 (9.5) | 4 (11.4) |
| GP | 34 (6.7) | 10 (15.9) | 1 (2.9) |
| Others | 35 (6.9) | 1 (1.6) | 1 (2.9) |
| EGFR/ALK-positive patients | Adenocarcinoma  (n = 303) | Squamous cell carcinoma  (n = 5) | Others  (n = 9) |
| TKIs | 157 (51.8) | 4 (80.0) | 4 (44.4) |
| PP | 119 (39.3) | 0 (0) | 4 (44.4) |
| TP | 17 (5.6) | 1 (20.0) | 1 (11.2) |
| Others | 10 (3.3) | 0 (0) | 0 (0) |
| Abbreviations: ALK, anaplastic lymphoma kinase; DP, docetaxcel with platinum; EGFR, epidermal growth factor receptor; GP, gemcitabine with platinum; mOS, median overall survival; PP, pemetrexed with platinum; TKIs, tyrosine kinase inhibitors; TP, paclitaxel with platinum. | | | |

**Figure S1. The OS of SM+BLT group stratified by Lung-molGPA. (A) Lung-molGPA 0-2; (B) Lung-molGPA 2.5-4.**

Abbreviations: BLT, brain localized treatment; BS, brain surgery; DBR, deferred brain radiotherapy; EBR, early brain radiotherapy; mOS, median overall survival.


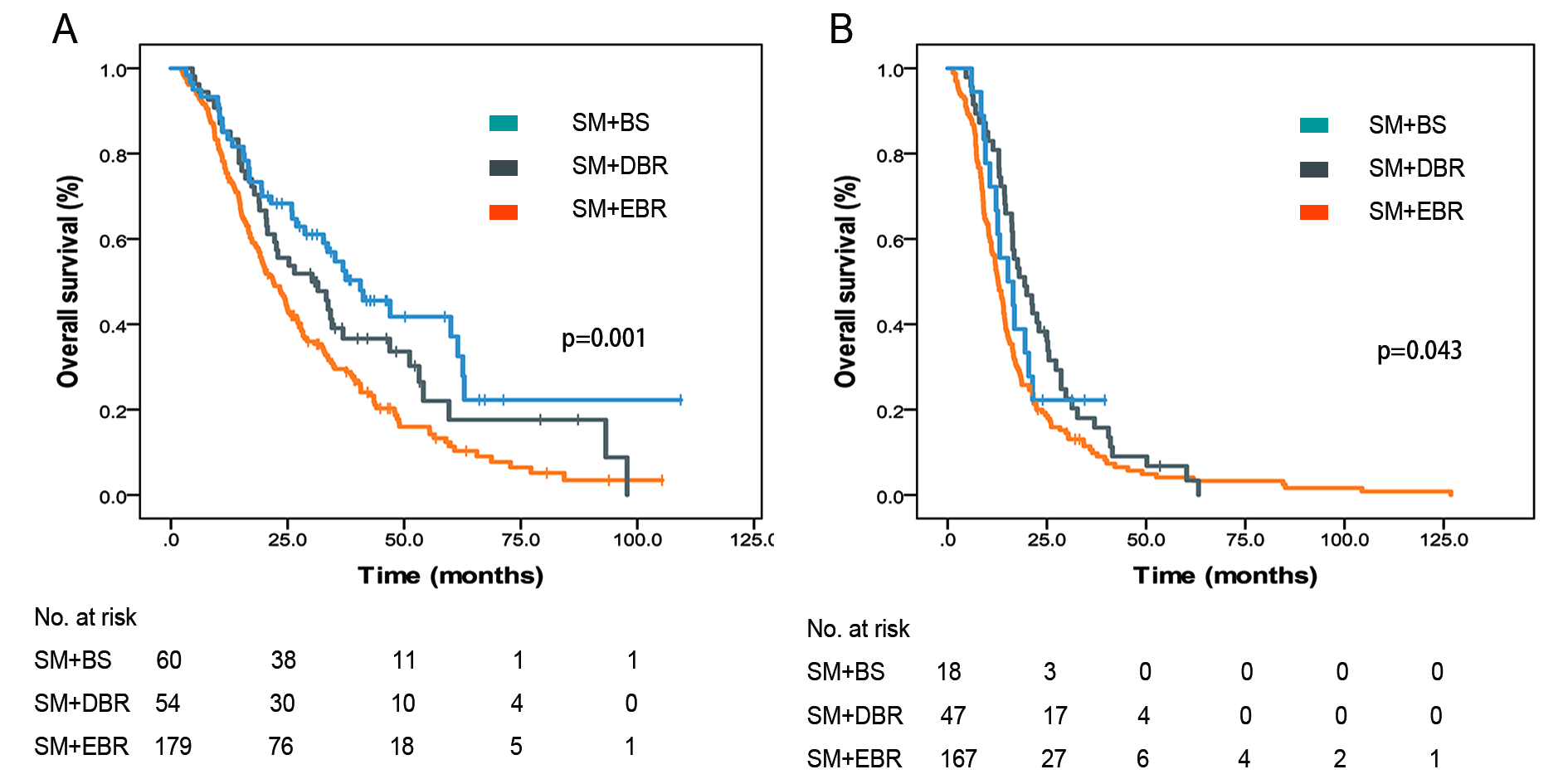


**Figure S2. Kaplan-Meier analysis comparing OS in patients treated with SM+EBR and SM+DBR after PSM. (A) In EGFR/ALK-negative/unknown cohort (1: 3 match). (B) In EGFR/ALK-positive cohort (1:1 match)**

Abbreviations: ALK, anaplastic lymphoma kinase; DBR, deferred brain radiotherapy; EBR, early brain radiotherapy; EGFR, epidermal growth factor receptor; mOS, overall survival.

**
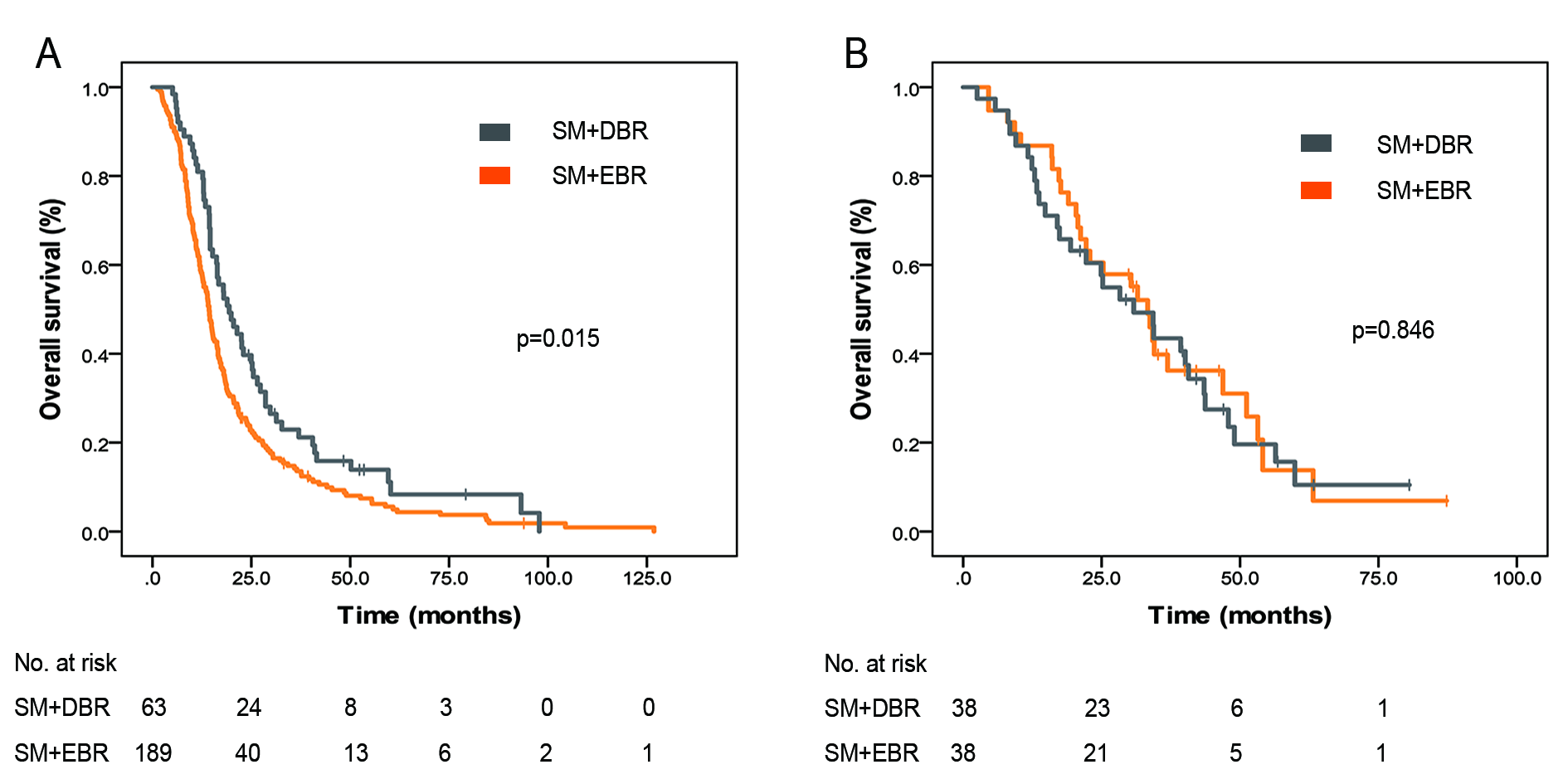
**
